# Effects of semaglutide on beta cell function and glycaemic control in participants with type 2 diabetes: a randomised, double-blind, placebo-controlled trial

**DOI:** 10.1007/s00125-017-4289-0

**Published:** 2017-05-19

**Authors:** Christoph Kapitza, Kirsten Dahl, Jacob B. Jacobsen, Mads B. Axelsen, Anne Flint

**Affiliations:** 1 Profil Institut für Stoffwechselforschung GmbH, Hellersbergstrasse 9, 41460 Neuss, Germany; 2 Novo Nordisk A/S, Søborg, Denmark

**Keywords:** Beta cell function, Glycaemic control, Placebo-controlled trial, Semaglutide, Type 2 diabetes

## Abstract

**Aims/hypothesis:**

Semaglutide is a glucagon-like peptide-1 analogue in development for the treatment of type 2 diabetes. Its effects on first- and second-phase insulin secretion and other measures of beta cell function and glycaemic control were assessed.

**Methods:**

In this single-centre, double-blind, placebo-controlled, parallel-group trial, conducted at the Profil Institut für Stoffwechselforschung, Germany, 75 adult (aged 18–64 years) participants with type 2 diabetes (eligibility: HbA_1c_ of 6.5–9.0% (47.5–74.9 mmol/mol); BMI 20.0–35.0 kg/m^2^; and treatment with diet and exercise and/or metformin monotherapy with a dose unchanged in the 30 days prior to screening) were randomised (1:1) to once-weekly s.c. semaglutide 1.0 mg (0.25, 0.5, 1.0 mg escalated) or placebo for 12 weeks. Co-primary endpoints were changes from baseline to end of treatment in the first (AUC_0–10 min_) and second (AUC_10–120 min_) insulin secretion phases, as measured by the IVGTT. An arginine stimulation test (AST) and a 24 h meal stimulation test were also conducted. A graded glucose infusion test (GGIT) assessed insulin secretion rate (ISR) in treated participants and a group of untreated healthy participants. Safety endpoints were also assessed.

**Results:**

In total, 37 participants received semaglutide and 38 received placebo. Following IVGTT, for insulin, both AUC_0−10min_ and AUC_10−120min_ were significantly increased with semaglutide (estimated treatment ratio [95% CI] 3.02 [2.53, 3.60] and 2.10 [1.86, 2.37], respectively; *p* < 0.0001). The 24 h meal test showed reduced fasting, postprandial and overall (AUC_0–24h_) glucose and glucagon responses with semaglutide (*p* < 0.0001). The AST showed that maximal insulin capacity increased following semaglutide treatment. During GGIT, semaglutide significantly increased ISR to levels similar to those in healthy participants. Semaglutide was well tolerated.

**Conclusions/interpretation:**

Twelve weeks of once-weekly treatment with semaglutide significantly improved beta cell function and glycaemic control in participants with type 2 diabetes.

**Trial registration:**

ClinicalTrials.gov NCT02212067

**Funding:**

The study was funded by Novo Nordisk A/S.

**Electronic supplementary material:**

The online version of this article (doi:10.1007/s00125-017-4289-0) contains peer-reviewed but unedited supplementary material, which is available to authorised users.

## Introduction

Beta cell dysfunction is a key component in the pathophysiology of type 2 diabetes [1]. Both the development and progression of type 2 diabetes are marked by declining beta cell mass and function. The UK Prospective Diabetes Study investigators estimated that 50% of beta cell function may already be lost by the time of diagnosis, and subsequent glycaemic deterioration has been found to be associated with continued loss of beta cell function [2]. Addressing beta cell function is critical to the durability of diabetes treatments [3]. Glucagon-like peptide-1 receptor agonists (GLP-1RAs) appear to have several beneficial effects on beta cell function, offering individuals with type 2 diabetes the potential for enduring glycaemic control [3]. GLP-1 has been shown to prevent glucolipotoxicity in beta cells, thereby helping to protect and improve their overall function [4].

GLP-1RAs help to restore beta cell sensitivity to elevated blood glucose and improve beta cell function across a number of indices in people with type 2 diabetes [5, 6]. These effects have been illustrated in a sequence of studies with the GLP-1 analogue liraglutide vs placebo. Liraglutide treatment resulted in increased first- and second-phase insulin secretion, increased maximal insulin secretion after arginine infusion [7–9], increased HOMA of beta cell function (HOMA-B), a higher level of C-peptide, and a reduced proinsulin-to-insulin ratio [10]. In another study, liraglutide restored beta cell responsiveness to glucose during a stepwise glucose clamp [11], which is in line with trials showing glucose-dependent insulin secretion [12], decreased glucagon secretion from pancreatic islets [12, 13] and delayed gastric emptying [14, 15].

Semaglutide (Novo Nordisk, Bagsværd, Denmark) is a GLP-1 analogue in development for the once-weekly treatment of type 2 diabetes. It has 94% structural homology to native human GLP-1, and is based on the same acetylation technology as liraglutide [16]. Important structural modifications make semaglutide more resistant to degradation by dipeptidyl peptidase-4 and improve binding to albumin [16]. These modifications result in a *t*_½_ of approximately 1 week, making semaglutide appropriate for once-weekly s.c. administration, with a fully retained potency [17].

This trial investigated the effects of semaglutide 1.0 mg at steady state (after 12 weeks of once-weekly administration) on different aspects of beta cell function in participants with type 2 diabetes. The trial included four tests that together provide a comprehensive picture of changes in beta cell function during treatment with semaglutide. The primary objective was to evaluate the effects of semaglutide on first- and second-phase insulin secretion. Secondary objectives were to investigate the effects of semaglutide on fasting and postprandial glucose, insulin, C-peptide and glucagon concentrations, and on maximal insulin secretory capacity. The beta cell responsiveness to graded glucose infusion was assessed in participants with type 2 diabetes treated with semaglutide or placebo and in a group of healthy untreated participants. Additionally, the study assessed the pharmacokinetics (PK), safety and tolerability of semaglutide.

## Methods

### Trial design

This was a single-centre, randomised, multiple-dose, double-blind, placebo-controlled, parallel-group trial in participants with type 2 diabetes. A healthy comparator group was also included specifically to evaluate beta cell responsiveness to graded glucose infusion. The trial was conducted at the Profil Institut für Stoffwechselforschung in Germany and complied with the International Conference on Harmonisation Good Clinical Practice guidelines [18] and the Declaration of Helsinki [19]. All participants provided written informed consent and the trial protocol was approved by German health authorities and local independent ethics committees.

**Fig. 1:**
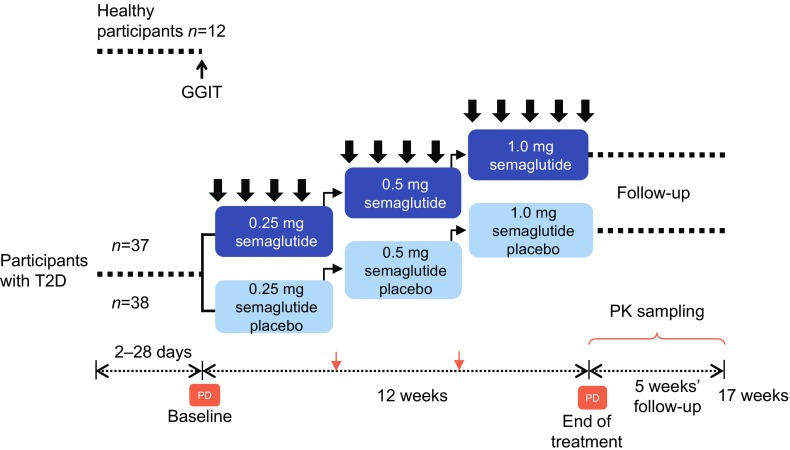
Trial design. Thick black arrows, study medication dose; thin black arrows, dose escalation; orange arrows, PK sampling; PD assessment comprised: IVGTT, AST, 24 h profiles and GGIT. T2D, type 2 diabetes

Participants with type 2 diabetes were randomised (1:1) to either once-weekly s.c. semaglutide 1.0 mg or placebo. Participants followed a fixed-dose escalation regimen (Fig. 1). Study medication (semaglutide, Novo Nordisk, or volume-matched placebo) was administered for 12 weeks, followed by a 5 week follow-up period. Healthy participants did not receive any treatment.

Semaglutide and placebo were visually identical and were packed blinded. The clinical study group, investigators and all other personnel and participants, except for staff conducting the treatment allocation, were blinded to the randomisation throughout the trial.

### Trial population

Participants were adults (aged 18–64 years). Additional eligibility criteria for participants with type 2 diabetes included: HbA_1c_ of 6.5–9.0% (47.5–74.9 mmol/mol); BMI of 20.0–35.0 kg/m^2^; and treatment with diet and exercise and/or metformin monotherapy with a dose unchanged in the 30 days prior to screening. At least 24 participants with type 2 diabetes of each sex were required to be enrolled. Participants in the healthy comparator group were required to have a BMI of 24.0–32.0 kg/m^2^ and an HbA_1c_ < 6.5% (47.5 mmol/mol). At least four healthy participants of each sex were required in the control group.

Key exclusion criteria for all participants included: any clinically significant disease history or systemic or organ disease; use of any prescription or non-prescription medication that would interfere with trial results, specifically systemic corticosteroids, non-selective beta blockers or thyroid hormones (unless thyroid hormone use was stable 2 months prior to screening).

Additional exclusion criteria for participants with type 2 diabetes included: impaired renal function (eGFR <60 ml min^−1^ 1.73 m^−2^ per the Modification of Diet in Renal Disease [MDRD] formula); recurrent severe hypoglycaemia; known proliferative retinopathy or maculopathy requiring acute treatment; treatment with glucose-lowering medication other than metformin 3 months prior to screening (except short-term insulin treatment in connection with intercurrent illness).

### Randomisation

Participants were always assigned the lowest available randomisation number. Allocation of treatment was performed by unblinded site staff according to the randomisation list.

### Study assessments

The IVGTT, arginine stimulation test (AST), graded glucose infusion test (GGIT) and meal stimulation over 24 h were conducted at baseline and end of treatment on different study days (Fig. 1).

The IVGTT evaluated the response to an i.v. bolus injection of 25 g glucose administered over 2 min. Blood samples for analysis of insulin, C-peptide, glucose and glucagon concentrations were drawn frequently from 30 min prior to glucose injection through to 2 h post-injection.

An AST was performed after the completion of the IVGTT to measure maximal insulin secretory capacity. An i.v*.* glucose infusion (150 mg/kg) was administered to achieve hyperglycaemia (target 16 mmol/l); 2 h later, 5 g arginine was administered i.v. over 30 s, and blood samples for the analysis of insulin, C-peptide, glucose and glucagon were drawn frequently until 35 min after arginine administration.

Fasting and postprandial glucose metabolism were evaluated over a 24 h period during which three standard meals (breakfast at 0 h, lunch at 5 h, high-protein dinner at 10 h) were served. Blood samples were drawn throughout this time to determine 24 h profiles for glucose, insulin, C-peptide and glucagon.

A GGIT was performed both in participants with type 2 diabetes and healthy participants to assess beta cell responsiveness. The glycaemic start level was 5 mmol/l (achieved by administration of i.v. glucose or insulin as required). The i.v. glucose infusion was adjusted to achieve sequential plasma glucose targets throughout the 3 h test of 5, 6, 7, 8, 9, 10, 11 and 12 mmol/l, with 25 min intervals between each target. Blood samples for analysis of insulin, C-peptide, glucose and glucagon levels were drawn throughout the test.

Adverse events (AEs) were recorded from the time of the participant signing informed consent through to the end of follow-up and were regarded as treatment-emergent adverse events (TEAEs) if beginning (or increasing in severity) after the first study dose and no later than the end of follow-up.

Blood samples were drawn throughout the trial to assess PK (trough values). Body weight was measured at all visits.

### Study endpoints

#### In participants with type 2 diabetes

The co-primary endpoints were the change from baseline to end of treatment (Week 12) in first- and second-phase insulin secretion, as measured during IVGTT (AUC_0−10min_ and AUC_10−120min_, respectively).

The secondary IVGTT-related pharmacodynamic (PD) endpoints were the change from baseline to end of treatment in AUC_0–10min_, AUC_10–120min_ and AUC_0–120min_ with respect to C-peptide, insulin secretion rate (ISR), glucagon and insulin (only AUC_0–120min_).

Secondary PD endpoints measured by the AST included the change from baseline to end of treatment in AUC_0–10min_ and AUC_0–30min_ for insulin, ISR and glucagon.

Secondary endpoints measured by meal stimulation included the change from baseline to end of treatment in the 24 h profiles of plasma glucose, glucagon, serum insulin and C-peptide, measured as total AUC_0–24h_ during a test day with three standardised meals. Change from baseline to end of treatment in meal-specific AUCs following breakfast, lunch and dinner (0–5 h, 5–10 h and 10–15 h, respectively) and the corresponding mean postprandial increments were added post hoc.

The change from baseline to end of treatment in body weight was an additional secondary endpoint.

Safety endpoints from baseline to follow-up included the number of TEAEs, the number of severe hypoglycaemic events (as defined by the ADA [20]) or blood glucose-confirmed (<3.1 mmol/l) hypoglycaemic events, and change from baseline to end of treatment in vital signs and standard laboratory test results.

#### In participants with type 2 diabetes and healthy participants

Graded glucose infusion-related secondary endpoints were change from baseline to end of treatment in the AUC of ISR and glucagon over the 5–12 mmol/l glucose level (AUC_5–12mmol,ISR_ and AUC_5–12mmol,glucagon_, respectively), and in the slope of the ISR vs glucose curve (dose–response relationship).

### PK endpoints

PK endpoints (AUC_0–168_, maximum concentration [C_max_], time to maximum concentration [t_max_], *t*_½_) were assessed for semaglutide 1.0 mg at steady state, based on blood sampled up to 840 h after the last dose. Additionally, trough semaglutide concentrations for each dose level at steady state (C_trough,SS_) were derived from blood samples drawn 1 week after the fourth dose at each of the three dose levels. Further information on PK endpoints is provided in the electronic supplementary material (ESM).

### Statistical analysis

Sample size was calculated based on the within-participant differences in AUC_0–17min,insulin_ and AUC_90–120min,insulin_ from an IVGTT in a crossover liraglutide study (data not published). A dropout rate of 13–15% was assumed. The enrolment of 37 participants in each treatment group was calculated to provide a combined power of 80.6% to detect a treatment difference corresponding to 20 nmol l^−1^ h^−1^ and 650 nmol l^−1^ h^−1^ for the first and second insulin secretion phases, respectively, assuming a corresponding SD of 23 nmol l^−1^ h^−1^ and 830 nmol l^−1^ h^−1^.

All AUCs were calculated by non-compartmental model-free methods using the linear trapezoidal method on the observations and actual time points.

The co-primary endpoints were analysed separately using linear normal models on log_10_-transformed data, including treatment as a fixed effect and baseline value as a covariate. Estimated treatment difference (ETD) was presented on the original scale as estimated treatment ratio (ETR) with 95% CIs and *p* value testing the null hypothesis of no treatment difference.

For all secondary AUC endpoints, the data were analysed and presented in the same way as for the primary endpoints.

The ISR was calculated from population-based C-peptide kinetic variables [21], using ISEC software (version 3.4a, September 1994), in which the insulin grid was set to equal the sample regimen, the basal measurement of C-peptide was included in the calculation and the error of C-peptide measurements was set to 5% [22]. The ISEC software can be obtained from the developer: Roman Hovorka, PhD, University of Cambridge, UK (https://www.scribd.com/document/177600095/Isec-Manual).

For the GGIT, the slope of the ISR vs glucose curve was calculated for each individual profile by a simple regression model on the log_10_-transformed data. Change from baseline was analysed in the same way as for the primary endpoints.

The mean postprandial increment was calculated as the incremental AUC (the total AUC–time curve minus the area under the pre-meal concentration during the same time interval) divided by the length of the time interval. The change from baseline to end of treatment in mean postprandial increment was analysed in a linear normal model on the original scale with treatment as a fixed effect and baseline value as a covariate.

Change from baseline in body weight was analysed using a mixed model for repeated measurements using all post-baseline data as the dependent variable, and with treatment, visit and treatment nested within visit as factors, and baseline body weight as a covariate. An unstructured covariance matrix was used to describe the variability for the repeated measurements for a participant.

## Results

The trial was conducted between 11 August 2014 and 11 May 2015. In total, 37 participants received semaglutide and 38 participants received placebo (ESM Fig. 1). Baseline characteristics were similar between the semaglutide and placebo groups (Table 1). All participants were white. Two participants receiving semaglutide withdrew from the trial, one due to a serious adverse event (SAE) during the follow-up period and one for other reasons in the middle of the treatment period (ESM Fig. 1). One participant receiving placebo discontinued as a result of a protocol violation prior to end of treatment.

**Table 1:** Demographics and baseline characteristics of study populations

	Semaglutide 1.0 mg *n* = 37	Placebo *n* = 38	Healthy *n* = 12
Age, years	56 (45–64)	57 (44–64)	43 (24–58)
HbA_1c_, %	7.3 (6.4–8.9)	7.3 (5.9–9.0)	N/A
HbA_1c_, mmol/l	56.1 (46.5–73.8)	55.7 (41.0–74.9)	N/A
Diabetes duration, years	8.3 (1.0–22.2)	8.7 (1.4–21.3)	N/A
Body weight, kg	93.2 (61.9–119.6)	90.0 (64.1–120.0)	81.9 (68.7–103.2)
BMI, kg/m^2^	29.5 (20.7–35.1)	29.7 (21.9–35.3)	26.8 (24.5–29.7)
Metformin use, *n* (%)	33 (89.1)	34 (89.5)	N/A
Male, *n* (%)	27 (73.0)	24 (63.2)	8 (66.7)

Data presented are mean (range) unless stated otherwise

N/A, not applicable

**Fig. 2:**
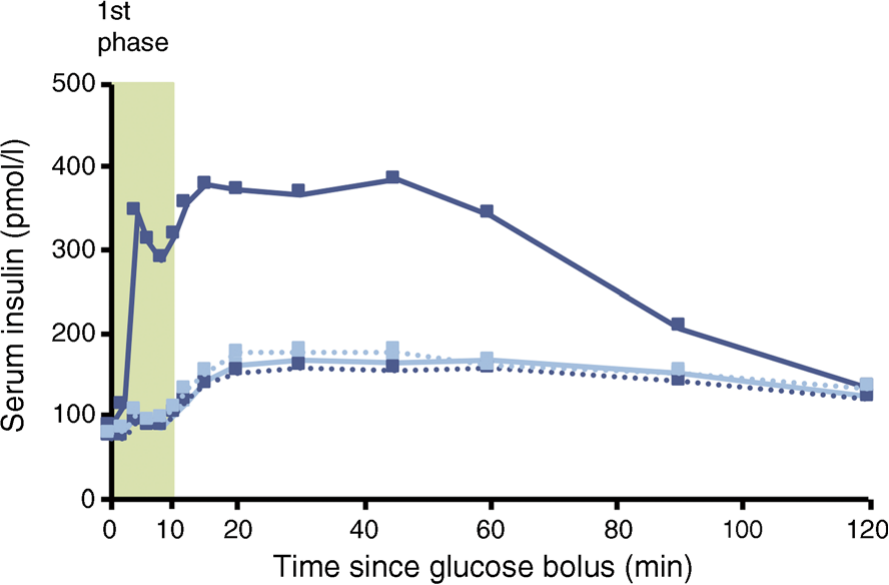
Geometric mean insulin response to an IVGTT in participants with type 2 diabetes before and after 12 weeks of treatment with semaglutide (*n* = 37, dark blue) or placebo (*n* = 37, light blue). One participant who received an incorrect glucose dose was excluded from all IVGTT analyses. Dotted lines represent baseline values and solid lines represent end of treatment values

**Fig. 3:**
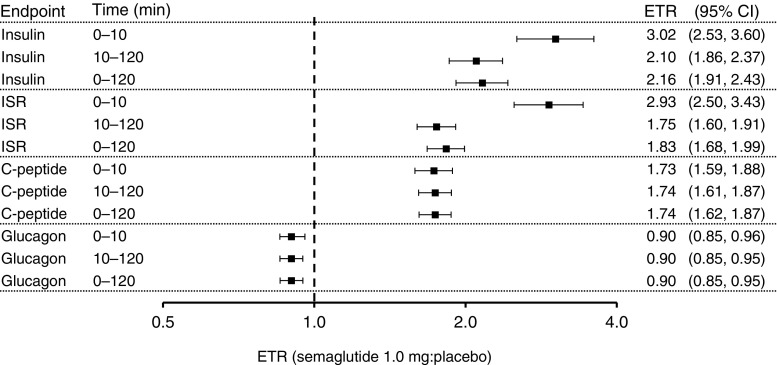
ETR (semaglutide:placebo) for endpoints from an IVGTT. Endpoints are the change from baseline to end of treatment in the AUC. One participant who received an incorrect glucose dose was excluded (*n* = 36 in both groups). 0–10 min, first phase; 10–120 min, second phase. All endpoints listed are change from baseline to end of treatment in AUC. All *p* values ≤0.0002

The geometric mean concentration–time profiles for insulin in participants with type 2 diabetes after an i.v. bolus injection of glucose are presented in Fig. 2. First-phase (AUC_0–10min,insulin_) and second-phase (AUC_10–120min,insulin_) insulin responses in the IVGTT were significantly increased after 12 weeks of treatment with semaglutide compared with placebo (ETR [95% CI] 3.02 [2.53, 3.60] and 2.10 [1.86, 2.37], respectively, both *p* < 0.0001; Fig. 3).

Changes from baseline to end of treatment in the AUC of ISR, C-peptide and glucagon in the IVGTT were also significantly greater in the semaglutide group compared with the placebo group (Fig. 3). The increases in the AUC of insulin and ISR were larger in the first phase than in the second phase of insulin secretion. The treatment effects of semaglutide in increasing the AUC of C-peptide and decreasing the AUC of glucagon were similar in each insulin secretion phase (Fig. 3). The magnitude of change in ISR was reflected in the reduction in glucose during the IVGTT (data not shown).

**Fig. 4:**
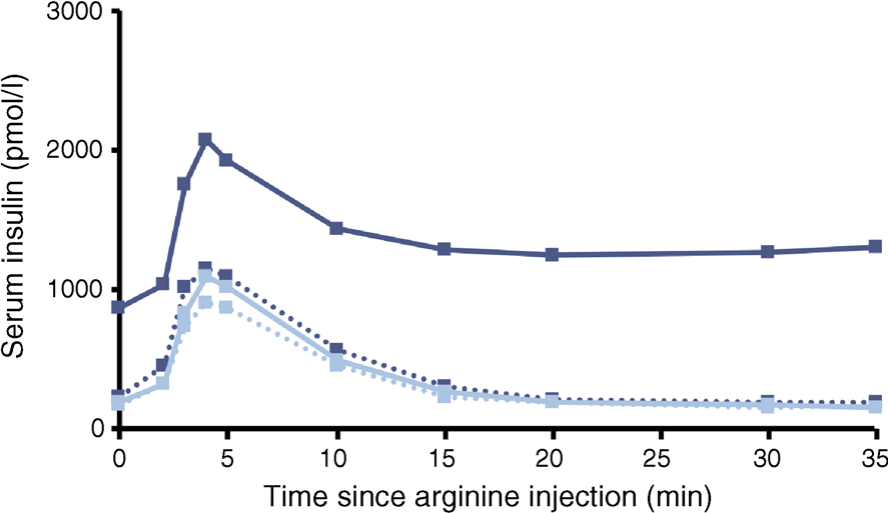
Serum insulin over time following hyperglycaemic ASTs in participants with type 2 diabetes before and after 12 weeks of treatment with semaglutide (*n* = 37, dark blue) or placebo (*n* = 38, light blue). Dotted lines represent baseline values and solid lines represent end of treatment values

Observed insulin levels during induced hyperglycaemic conditions immediately prior to the administration of arginine at end of treatment were higher for the semaglutide group than for the placebo group (Fig. 4). The insulin response to arginine was significantly larger after semaglutide treatment compared with placebo (ETR [95% CI]: AUC_0–10min_ 2.82 [2.39, 3.32], AUC_0–30min_ 4.42 [3.74, 5.22]; *p* < 0.0001 for both). A larger ISR response was also observed after semaglutide treatment vs placebo (ETR [95% CI]: AUC_0–10min_ 1.69 [1.49, 1.92], AUC_0–30min_ 2.69 [2.38, 3.05]; *p* < 0.0001 for both). The glucagon response to arginine was significantly lower with semaglutide treatment vs placebo (ETR [95% CI]: AUC_0–10min_ 0.80 [0.75, 0.87], AUC_0–30min_ 0.82 [0.78, 0.87]; *p* < 0.0001 for both). As can be seen from both the AUC_0–10min_ and the AUC_0–30min_ results, these differences between treatment groups occurred throughout the duration of the AST.

**Fig. 5:**
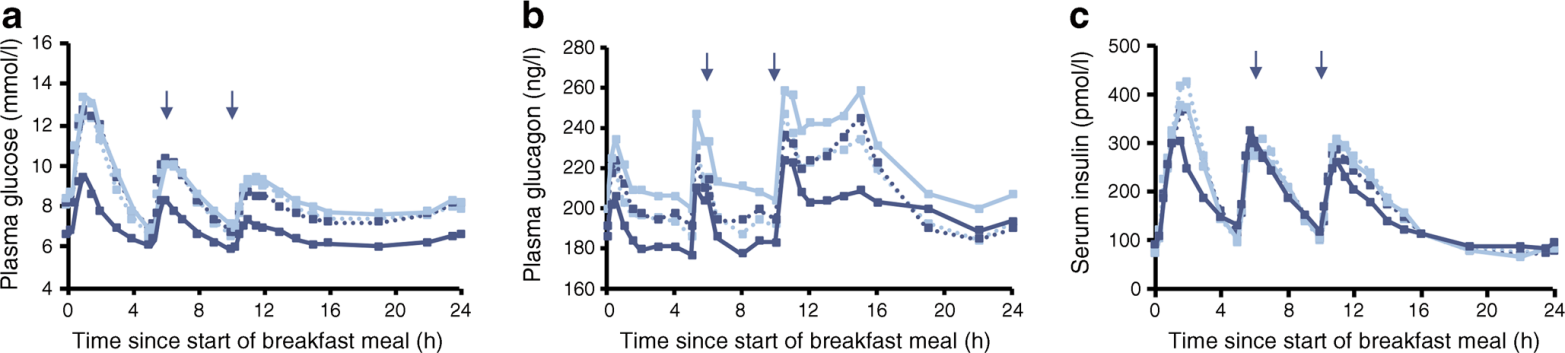
Plasma glucose (**a**), plasma glucagon (**b**) and serum insulin (**c**) 24 h profiles in participants with type 2 diabetes before and after receiving 12 weeks of treatment with semaglutide (*n* = 37, dark blue) or placebo (*n* = 38, light blue). Dotted lines represent baseline values and solid lines represent end of treatment values. Arrows represent the start of meals

In the 24 h meal test, compared with placebo, AUC_0–24h_ for glucose and glucagon was significantly reduced after semaglutide treatment (ETR [95% CI]: 0.78 [0.74, 0.82] and 0.88 [0.83, 0.93], respectively; *p* < 0.0001 for both), while AUC_0–24h_ for insulin had no treatment effect (ETR [95% CI]: 1.01 [0.93, 1.10]; *p* = 0.8243; Table 2). C-peptide values were significantly increased after semaglutide treatment (ETR [95% CI]: 1.05 [1.00, 1.10]; *p* < 0.0458; Table 2). The decrease of glucose and glucagon was observed at the first sampling point, which was in fasting conditions, and was sustained throughout the duration of the test (Fig. 5).

**Table 2:** Meal stimulation test. Semaglutide: placebo differences in the change from baseline to end of treatment AUC for glucose, glucagon, insulin and C-peptide, shown as absolute fasting and postprandial AUC, and mean postprandial increments

	Fasting state	Breakfast	Lunch	Dinner	Overall
Absolute fasting and postprandial response, ETR (95% CI)		AUC_0–5h_	AUC_5–10h_	AUC_10–15h_	AUC_0–24h_
Glucose	0.78 (0.74, 0.83)	0.71 (0.67, 0.76)	0.79 (0.74, 0.85)	0.80 (0.75, 0.86)	0.78 (0.74, 0.82)
Glucagon	0.92 (0.86, 0.99)	0.86 (0.82, 0.91)	0.86 (0.81, 0.91)	0.85 (0.79, 0.91)	0.88 (0.83, 0.93)
Insulin	1.30 (1.11, 1.53)	0.95 (0.84, 1.07)	1.05 (0.94, 1.18)	0.90 (0.81, 1.01)	1.01 (0.93, 1.10)
C-peptide	1.23 (1.14, 1.32)	1.03 (0.96, 1.10)	1.06 (0.99, 1.14)	0.97 (0.91, 1.04)	1.05 (1.00, 1.10)
Mean postprandial increment, ETD (95% CI)	–	iAUC_0–5h_/5 h	iAUC_5–10h_/5 h	iAUC_10–15h_/5 h	–
Glucose, mmol/l	N/A	−1.11 (−1.52, −0.71)	−0.92 (−1.30, −0.54)	−0.60 (−0.93, −0.28)	N/A
Glucagon, ng/l	N/A	−12.86 (−23.38, −2.34)	−5.82 (−13.86, 2.23)	−11.49 (−21.91, −1.06)	N/A
Insulin, pmol/l	N/A	−43.47 (−73.06, −13.89)	−15.33 (−50.80, 20.15)	−42.90 (−81.81, −3.99)	N/A
C-peptide, nmol/l	N/A	−0.192 (−0.316, −0.067)	−0.086 (−0.229, 0.056)	−0.275 (−0.423, −0.128)	N/A

iAUC, incremental AUC; N/A, not applicable

In post hoc analyses (Table 2), fasting glucose (ETR [95% CI]: 0.78 [0.74, 0.83]) and glucagon (0.92 [0.86, 0.99]) was shown to decrease, while insulin (1.30 [1.11, 1.53]) and C-peptide (1.23 [1.14, 1.32]) increased after treatment with semaglutide vs placebo. In the individual meal periods, assessed as change from baseline to end of treatment, semaglutide significantly decreased postprandial glucose and glucagon responses compared with placebo, but did not affect insulin and C-peptide. For glucose, following the three meals, the absolute postprandial response was lowered by 20–29% and mean postprandial increments were lowered by 0.6–1.1 mmol/l for semaglutide compared with placebo. For glucagon, the absolute postprandial response after breakfast and dinner was lowered by 14–15% and postprandial increments were lowered by approximately 12 ng/l compared with placebo; however, the treatment difference following lunch was not statistically significant. For insulin and C-peptide, the absolute postprandial responses were similar during all three meals for semaglutide and placebo, while the mean postprandial increments were lowered following breakfast and dinner. In the post-lunch period the treatment differences were not statistically significant.

In the GGIT, both the increase in ISR AUC over the 5–12 mmol/l glucose interval (AUC_5–12mmol_) from baseline to end of treatment and the increase in the corresponding slope of the ISR vs glucose curve (dose–response relationship) were significantly greater in the semaglutide group than in the placebo group (ETR [95% CI]: 2.45 [2.16, 2.77] and 2.78 [2.44, 3.16], respectively; *p* < 0.0001 for both). Changes in glucagon levels with semaglutide treatment (AUC_5–12mmol_) were significantly lower compared with placebo (0.87 [0.82, 0.93]; *p* < 0.0001).

**Fig. 6:**
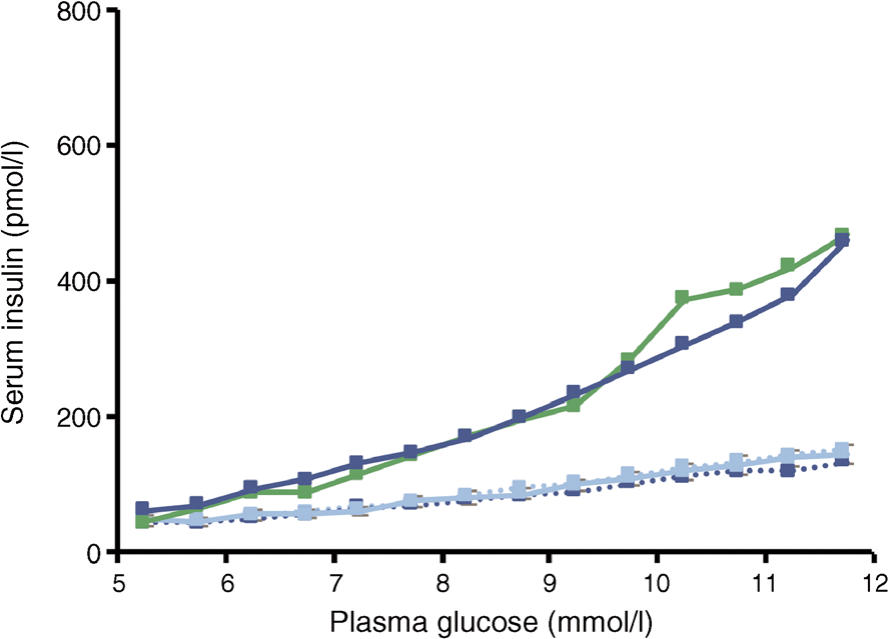
Insulin response to a graded glucose infusion in participants with type 2 diabetes before and after receiving 12 weeks of treatment with semaglutide (*n* = 37, dark blue) or placebo (*n* = 38, light blue), and in healthy participants (*n* = 12, green). Dotted lines represent baseline values and solid lines represent end of treatment values

The end of treatment values of these variables (AUC_5–12mmol_ for ISR, glucagon and the slope of the ISR vs glucose curve) in the semaglutide group were more similar to those for the healthy participants than for the participants receiving placebo, especially for the ISR and the slope of the ISR–glucose concentration curve (Table 3, Fig. 6).

**Table 3:** Endpoints from GGIT test after 12 weeks of treatment

	Semaglutide 1.0 mg *n*=36^a^	Placebo *n*=37^b^	Healthy participants, no treatment *n* = 12
ISR AUC_5–12mmol_, pmol/kg	43.9 (44.6)	19.5 (43.3)	45.7 (31.0)
Slope ISR vs glucose, pmol × l/(min mmol^−1^ kg^−1^)	1.3 (53.7)	0.6 (41.2)	1.4 (35.8)
Glucagon AUC_5–12mmol_, ng/l	994.9 (17.4)	1129.4 (19.2)	875.9 (12.9)

Data are presented as geometric mean (coefficient of variance, %)

^a^One participant was withdrawn for ‘other reason’ prior to the end of treatment visit and was not included in this analysis

^b^One participant discontinued prior to the end of treatment visit and was not included in this analysis

Body weight decreased by 4.2 kg in the semaglutide group, compared with 0.1 kg for placebo (ETD [95% CI] −4.1 [−5.1, −3.1]) over 12 weeks.

The PK data for semaglutide were consistent with those previously reported. PK values, including trough values during dose escalation, were indicative of treatment compliance (ESM Fig. 2 and ESM Table 1).

TEAEs were reported in 75.7% of participants receiving semaglutide and 55.3% receiving placebo (ESM Table 2). The most common type of TEAE in the semaglutide group, and thus the primary reason for the difference in incidence of TEAEs between the treatment groups, was gastrointestinal, reported in 19 (51.4%) participants in the semaglutide group and six (15.8%) participants in the placebo group. Of these, the most common events were nausea, vomiting and diarrhoea, reported in seven (18.9%), six (16.2%) and four (10.8%) semaglutide-treated participants, respectively, and in two (5.3%), two (5.3%) and three (7.9%) placebo-treated participants, respectively. Most AEs were mild to moderate in severity and transient in nature. One TEAE in the semaglutide group with a fatal outcome (pedestrian traffic accident) led to participant withdrawal. The investigator assessed the event as unlikely to be related to treatment. Two other TEAEs classed as serious occurred: one instance of angina pectoris and one hypertensive emergency. In both instances, the event resolved and the participant recovered.

## Discussion

This study investigated the effects of 12 weeks of once-weekly s.c. semaglutide treatment on various aspects of beta cell function in participants with type 2 diabetes. It was demonstrated by IVGTT that semaglutide treatment increased first- and second-phase insulin secretion threefold and twofold, respectively, compared with placebo. Correspondingly, levels of glucagon and glucose were decreased with semaglutide vs placebo. Similar findings have been reported for once-daily liraglutide, which significantly increased both first- and second-phase insulin secretion after 14 weeks of treatment in participants with type 2 diabetes [7].

Results from the AST under hyperglycaemic conditions showed that maximal insulin capacity had improved following semaglutide treatment. Despite insulin levels prior to the test being higher in semaglutide-treated participants than in participants receiving placebo, insulin levels increased immediately in response to the stimulus and remained high for the duration of the test. This effect could contribute to the reported efficacy of semaglutide in improving glycaemic control [23], particularly as recent research suggests that individuals with sustained endogenous insulin-secreting capacity may benefit more from GLP-1RA therapy [24].

In the 24 h meal test, encompassing three standardised meals, semaglutide reduced glucose and glucagon, and increased C-peptide levels (a marker of endogenous insulin production) in comparison with placebo. Reductions in glucose and glucagon levels with semaglutide were observed in the fasting as well as in the postprandial state, both in terms of the absolute levels and the incremental response. As shown with other GLP-1RAs, a higher insulin level was observed in the fasting state after semaglutide treatment despite lower fasting glucose levels, possibly due to the well-known incretin effect of the GLP-1 class [3, 15]. Notwithstanding this higher initial insulin level, similar postprandial insulin profiles were observed with semaglutide treatment and placebo. The similar insulin profiles were probably related to the lower incremental increase in glucose. A potential additional factor explaining this observation may be the loss of body weight during the course of the trial, possibly impacting insulin resistance. These findings are consistent with previous observations on the relative contributions of insulin stimulation and glucagon inhibition in overall glucose turnover mediated by exogenous GLP-1 and other GLP-1RAs [25]. Mean 2 h postprandial glucose was lower than the 2 h postprandial goal (<10 mmol/l) as recommended by the ADA [26], and more than 80% of the semaglutide-treated participants achieved the ADA target.

Beta cell responsiveness was markedly increased after treatment with semaglutide compared with placebo, and at the end of treatment closely resembled that of healthy participants, as measured by the GGIT. It is important to note, however, that BMI was higher in participants with type 2 diabetes than in healthy participants; differences in fat mass could potentially influence insulin sensitivity and this should be kept in mind when interpreting these results [27]. In addition, mean age was also higher in participants with type 2 diabetes than in healthy participants; the negative impact of ageing on beta cell responsiveness, triacylglycerol levels [28] and glucose homeostasis should also be considered in this context [29]. The possible role of managing beta cell function in type 2 diabetes, as a means to delay disease progression, has been a subject of discussion among researchers [30]. GLP-1RAs have attenuated beta cell dysfunction to varying degrees both in animal models [31] and in humans with type 2 diabetes [32].

Semaglutide treatment was associated with marked weight loss, even during this relatively short trial. These weight loss effects have persisted in trials of longer durations with semaglutide [33] and other GLP-1RAs [34, 35]. The high proportion of individuals with type 2 diabetes who are overweight or obese makes the combination of glycaemic control and body weight loss highly relevant to clinical practice [36].

No new safety or tolerability issues were observed for semaglutide. Most AEs were mild or moderate in severity and there were no serious TEAEs that were considered likely to be related to treatment. Longer and larger scale clinical trials will provide more information on the safety profile of semaglutide [23]. As reported with other GLP-1RAs [37, 38], gastrointestinal disorders were the most commonly reported AEs with semaglutide treatment. The incidence of such events has been shown to diminish over time with liraglutide [38, 39] and can be partially ameliorated with dose escalation at the start of treatment [17].

This study was limited by its relatively small size and short duration. The SUSTAIN clinical trial programme, comprising six global phase 3 trials, will determine how the improvements in beta cell function with semaglutide translate into clinical benefits, compared with current treatment options.

In conclusion, the results of the current study are consistent with previous findings during treatment with the GLP-1 analogue liraglutide [25], and suggest that treatment with semaglutide may offer a protective effect on beta cell function. In addition, the results show that semaglutide is a promising once-weekly GLP-1 analogue for the treatment of type 2 diabetes, associated with improved beta cell responsiveness comparable with that observed in healthy individuals. All aspects of beta cell function, including first- and second-phase insulin responses, were significantly increased in participants with type 2 diabetes treated with semaglutide vs placebo after 12 weeks of treatment. In addition, semaglutide reduced fasting and postprandial glucose and glucagon levels, compared with placebo, in a 24 h meal test. Improved glycaemic responses appeared to be a combined effect on the pancreas of increased insulin secretion and decreased glucagon response. Semaglutide was well tolerated.

## Electronic supplementary material

ESM (PDF 74 kb)

## Abbreviations

AE: Adverse event
AST: Arginine stimulation test
C_max_: Maximum concentration
ETR: Estimated treatment ratio
GGIT: Graded glucose infusion test
GLP-1RA: Glucagon-like peptide-1 receptor agonist
HOMA-B: HOMA of beta cell function
ISR: Insulin secretion rate
MDRD: Modification of Diet in Renal Disease
PD: Pharmacodynamic
PK: Pharmacokinetic
SAE: Serious adverse event
TEAE: Treatment-emergent adverse event
t_max_: Time to maximum concentration
