# Supplementary material for: Effects of semaglutide on beta cell function and glycaemic control in participants with type 2 diabetes: a randomised, double-blind, placebo-controlled trial
Source: Diabetologia. 2017 May 19;60(8):1390–9. doi: 10.1007/s00125-017-4289-0 (PMC5491562; doi:10.1007/s00125-017-4289-0)

Effects of semaglutide on beta cell function and glycaemic control in participants with type 2 diabetes: a randomised, double-blind, placebo-controlled trial

Electronic Supplementary Materials

Pharmacokinetic analyses

The following steady-state semaglutide PK endpoints were derived: AUC_{0-168} (area under the time–concentration curve during a dosing interval), calculated using standard non-compartmental methods using the linear trapezoidal method on the observed concentrations using actual time points; maximum concentration (C_{max}), derived as the maximum of all valid concentrations of semaglutide from 0 to 168 h; time to maximum concentration (t_{max}); half-life ($t_{½}$), calculated as $log_2/\lambda z$, where λz was estimated by log-linear regression on the terminal part of the semaglutide concentration–time curve; total apparent plasma clearance of semaglutide (CL/F), calculated as dose/AUC_{0-168}; volume of distribution (Vz/F), calculated as (CL/F)/λz.

ESM Table 1. Semaglutide trough concentrations* and PK endpoints at steady state

	**Semaglutide 0.25 mg N=37**	**Semaglutide 0.5 mg N=36**	**Semaglutide 1.0 mg N=36**
**Trough concentrations**			
**$C_{min,semaglutide}$ (nmol/l)**<br>Geometric mean (CV, %)	6.1 (27.4)	11.7 (26.4)	22.8 (23.9)
**PK endpoints**			
**$AUC_{0–168h}$ (nmol l^{-1} h)**<br>Geometric mean (CV, %)<br>min, max	N/A	N/A	4684 (18.8)<br>2515, 7956
**C_{max} (nmol/l)**<br>Geometric mean (CV, %)<br>min, max	N/A	N/A	32.2 (19.1)<br>16.8, 55.1
**t_{max} (h)**<br>Median<br>min, max	N/A	N/A	36.0<br>4.0, 165.0
**$t_{½}$ (h)**<br>Geometric mean (CV, %)<br>min, max	N/A	N/A	149 (10.9)<br>126, 189
**CL/F (l/h)**<br>Geometric mean (CV, %)<br>min, max	N/A	N/A	0.052 (18.8)<br>0.031, 0.970
**V_z/F (l)**<br>Geometric mean (CV, %)<br>min, max	N/A	N/A	11.2 (18.6)<br>7.3, 17.8

*1 week after the fourth dose of 0.25 mg, 0.5 mg and 1.0 mg semaglutide administration. AUC, area under the time–concentration curve; C_{max}, maximum concentration; CL/F, total plasma clearance; $C_{min,semaglutide}$, trough semaglutide plasma concentration; CV, coefficient of variance; PK, pharmacokinetic; t_{max}, time to maximum concentration; N/A, not applicable; $t_{½}$, half-life; V_z/F, volume of distribution. $t_{½}$ and Vz/F could only be estimated in 34 participants.

Adverse events

ESM Table 2. TEAEs

	Semaglutide N=37	Placebo N=38	Total N=75
	Number of participants with events (%)		
Any TEAE	28 (75.7)	21 (55.3)	49 (65.3)
Serious TEAE	2 (5.4)	0	2 (2.7)
Death*	1 (2.7)	0	1 (1.3)
TEAE leading to withdrawal	1 (2.7)	0	1 (1.3)
Severe TEAE	1 (2.7)	0	1 (1.3)
TEAEs occurring in >2 participants			
Gastrointestinal disorders	19 (51.4)	6 (15.8)	25 (33.3)
Infections and infestations	13 (35.1)	8 (21.1)	21 (28.0)
Nervous system disorders	5 (13.5)	7 (18.4)	12 (16.0)
Metabolism and nutrition disorders	4 (10.8)	1 (2.6)	5 (6.7)
Musculoskeletal and connective disorders	1 (2.7)	4 (10.5)	5 (6.7)
Vascular disorders	3 (8.1)	2 (5.3)	5 (6.7)
Injury, poisoning and procedural complications	1 (2.7)	2 (5.3)	3 (4.0)

*Road traffic accident regarded unlikely to be related to study drug. TEAE, treatment-emergent adverse events. There were no clinically relevant conditions reported for vital signs. Systolic blood pressure slightly decreased throughout the course of the treatment period in participants on semaglutide. The mean pulse increased shortly after initiation of treatment with semaglutide, and peaked after the first dose of 0.5 mg semaglutide (change from baseline geometric mean was 5 beats per min [SD 10]). Subsequently, the mean pulse decreased, and at last follow-up visit it returned to a level similar to that observed prior to treatment start. There were no TEAEs from clinical laboratory results and no anti-semaglutide antibodies were detected in this study.

ESM Fig. 1. Participant flow. AE, adverse event; T2D, type 2 diabetes; TEAE, treatment-emergent adverse event

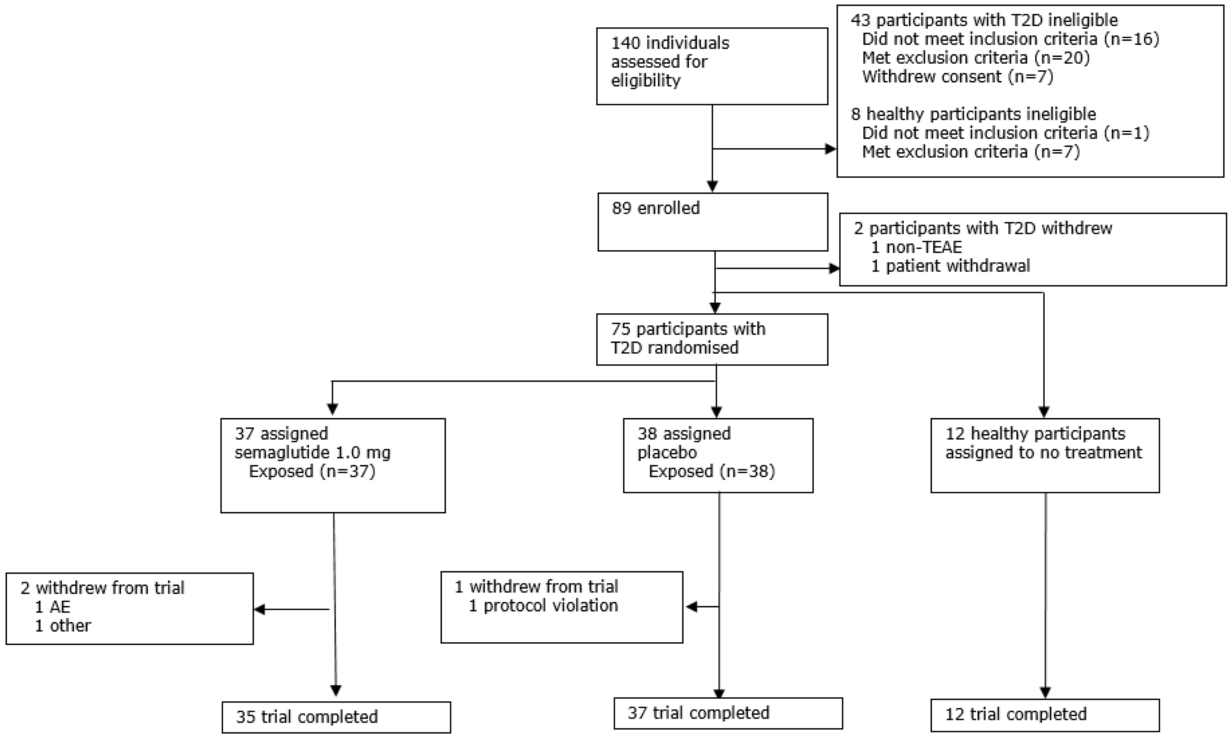

ESM Fig. 2. Semaglutide profile at steady state (logarithmic scale). Dashed line represents reference line for lower limit of quantification

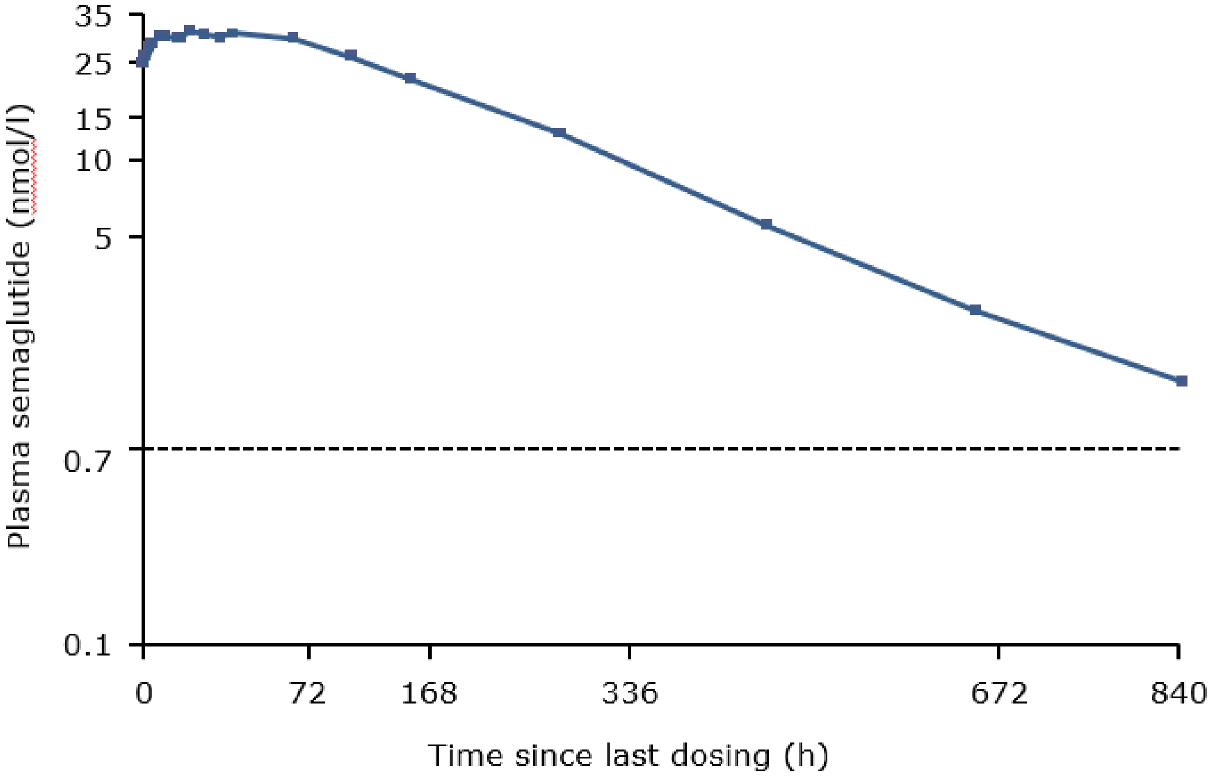

Supplement: Supplementary file 1 — (PDF 74 kb) [file 125_2017_4289_MOESM1_ESM.pdf]
